# Weekend effect: a great problem with a potential solution

**DOI:** 10.1186/s13054-017-1864-1

**Published:** 2017-11-17

**Authors:** Ana Abella, Beatriz Lobo-Valbuena, Marcela Hómez, Federico Gordo

**Affiliations:** 1 Intensive Care Unit, Hospital Universitario del Henares, Avenida María de Curie s/n. 28822, Coslada, Madrid Spain; 2 Universidad Francisco de Vitoria, Facultad Ciencias de la Salud, Edificio E, Ctra. M-515 Pozuelo-Majadahonda, Madrid, Spain

We have read with great interest the article by Zajic et al. [1] showing that patients admitted to the intensive care unit (ICU) on weekends had a higher mortality rate.

Recently, in a study of by group we found similar results; factors independently associated with hospital mortality were the Simplified Acute Physiology Score (SAPS)3 (odds ratio (OR) 1.10; 95% confidence interval (CI) 1.08–1.12) and belonging to the “off-hours” group (weekend and holidays; OR 2.00; 95% CI 1.20–3.33) [2].

We firmly believe in the priority of early detection of patients at risk of poor outcome, not only of the so-called “time-dependent” pathologies such as sepsis, stroke or ischemic heart disease, where early intervention can improve prognosis and reduce onset complications, but also including all potential ICU patients. For this reason, we have implemented since 2008 the “ICU without walls” protocol, which allows early detection of high-risk patients using a computer data alert system based on laboratory data [3].

With the assumption that worse prognosis may be related to the late detection of these patients, the “ICU without walls” protocol was developed also during weekends. Through a multivariate analysis, SAPS3 (OR 1.08, 95% CI 1.06–1.11) and being part of the intervention group (OR 0.33, 95% CI 0.12–0.89) were independently associated with mortality [4].

We would also like to emphasize that in the publication by Zajic and co-authors [1], patients admitted after scheduled surgery presented a lower ICU death risk (hazard ratio 0.43; 95% CI 0.39–0.46). Not withdrawing scheduled surgery patients or not differentiating them from unplanned/urgent admissions could imply a selection bias, despite a baseline mortality risk adjustment using the SAPS3. Moreover, the difference in reasons for ICU admission between weekdays and weekends is noteworthy, as Zajic et al. discuss.

Finally, we would like to point out that the early detection of patients at risk of organ failure is a task that must be adapted to the circumstances of each center. A multidisciplinary team coordinated by the intensivist, with the help of electronic and organizational resources, may help to resolve this issue. Regarding this topic, Durie et al. [5] demonstrate good clinical results after applying a multidisciplinary approach, named “Code-ICU”, dedicated to properly identifying candidates for ICU admission, displaying a reduction in ICU length of stay and hospital length of stay.

## Abbreviations

ICU: Intensive care unit
OR: Odds ratio
SAPS: Simplified Acute Physiology Score
